# Exploring the Link Between IL-6 rs1800795 G > C SNP and the Severity of Epstein-Barr Virus-Associated Multiple Sclerosis: Potential Impact on Cognitive Impairment

**DOI:** 10.1007/s12035-025-05211-x

**Published:** 2025-07-15

**Authors:** Tokka M. Hassan, Azza M. El Amir, Nahla Elsayed Nagy, Nashwa El-Khazragy

**Affiliations:** 1 Egypt Center for Research and Regenerative Medicine (ECRRM), Cairo, 11599 Egypt; 2 Department of Biotechnology, Faculty of Science, Cairo University, Giza, 12613 Egypt; 3 Department of Neuropsychiatry, Faculty of Medicine, Ain Shams University, Cairo, 11566 Egypt; 4 Department of Clinical Pathology-Hematology and AinShams Medical Research Institute (MASRI), Faculty of Medicine, Ain Shams University, Cairo, 11566 Egypt

**Keywords:** Multiple sclerosis, Cognitive disability, IL-6 rs1800795 G > C, EBV

## Abstract

Multiple Sclerosis (MS) is a chronic immune-mediated neurological disorder frequently accompanied by cognitive impairment, which affects up to 60% of patients and is associated with faster disease progression and greater disability. Interleukin-6 (IL-6), a key proinflammatory cytokine involved in neuroinflammation, has been implicated in MS pathogenesis, and the rs1800795 (−174 G>C) single nucleotide polymorphism (SNP) in the IL6 gene may influence disease susceptibility and clinical severity. This study investigated the association between the IL6 rs1800795 polymorphism and clinical outcomes in Epstein-Barr virus (EBV)-positive MS patients, with a particular focus on cognitive dysfunction. A case-control design was employed, including 300 participants: 150 EBV-positive MS patients and 150 matched healthy controls. Genotyping was performed using TaqMan-based PCR, and clinical data such as disability status, disease progression, and cognitive performance were analyzed. The CC genotype was significantly more frequent in MS patients and was associated with a higher risk of severe disability (OR = 6.11, *p* = 0.0004), faster disease progression, and increased likelihood of cognitive impairment. These findings suggest that the IL6 rs1800795 polymorphism, particularly the CC genotype, contributes to MS susceptibility and adverse clinical outcomes. IL6 genotyping may hold promise as a predictive tool for disease progression and cognitive decline in EBV-associated MS, offering insights for more personalized therapeutic strategies.

## Introduction

Multiple sclerosis (MS) is a chronic, immune-mediated demyelinating disease of the central nervous system (CNS) that primarily affects young adults, with a peak onset between 20 and 40 years of age [1]. Globally, MS affects more than 2.8 million people, with incidence rates varying significantly by geography, ethnicity, and gender being two to three times more common in women than men [2, 3]. The disease is characterized by inflammation, demyelination, and neurodegeneration, leading to a wide spectrum of clinical manifestations, including visual disturbances, motor dysfunction, fatigue, and cognitive decline [4]. Cognitive impairment in MS, including deficits in memory, attention, executive functions, and processing speed, contributes substantially to loss of quality of life and social functioning [5, 6]. Pathogenetically, MS is thought to arise from a complex interplay between genetic susceptibility and environmental triggers [7], leading to an aberrant autoimmune response directed against myelin and other CNS antigens [8]. The natural course of MS can be classified into relapsing-remitting MS (RRMS), secondary progressive MS (SPMS), and primary progressive MS (PPMS), each with distinct trajectories of disease progression and prognosis [9].

The treatment of MS has evolved substantially in the past two decades, transitioning from broad-spectrum immunosuppressants to targeted disease-modifying therapies (DMTs) that modulate specific immune pathways [10]. Despite these advances, treatment response remains highly variable among individuals, underscoring the role of underlying genetic and environmental heterogeneity in modulating disease course and therapeutic outcomes [11].

Emerging evidence implicates both environmental and genetic factors in the pathogenesis and progression of MS. Epstein-Barr virus (EBV) infection, in particular, has gained recognition as a critical environmental trigger for MS. [7] Epstein-Barr virus (EBV) seropositivity is observed in over 95% of individuals with MS, compared to significantly lower rates in the general population. This near-universal prevalence supports the growing evidence that EBV plays a critical role in MS pathogenesis [12]. The mechanisms by which EBV may promote MS are multifactorial and likely involve molecular mimicry, chronic CNS inflammation, and dysregulation of immune responses [13].

Concurrently, genetic predisposition plays a pivotal role in modulating MS susceptibility and disease course [14]. Polymorphisms in genes involved in immune regulation have been repeatedly associated with MS. [15] Among these, interleukin-6 (IL-6), a pleiotropic cytokine with roles in inflammation, immunity, and CNS homeostasis, has garnered considerable attention. IL-6 promotes B-cell maturation, T-cell differentiation, and acute-phase responses, processes that are intricately involved in MS pathophysiology [16]. Aberrant IL-6 signaling has been implicated in neuroinflammatory cascades, leading to demyelination and neurodegeneration [17].

A particularly interesting focus is the IL6 174 G>C rs1800795 single nucleotide polymorphism (SNP) in the IL-6 gene promoter region, resulting in a G to C substitution. This polymorphism is functional, influencing IL-6 transcriptional activity and serum levels. Specifically, the C allele has been associated with higher basal and stimulated IL-6 expression compared to the G allele [18]. Given IL-6's dual roles in both peripheral and CNS immune responses, variations in its expression levels could significantly impact MS severity and its clinical outcomes, including cognitive impairment [17, 19, 20].

Despite extensive research linking IL-6 to MS in general [21], few studies have specifically explored the interaction between IL6-174 G>C rs1800795 polymorphism [18], EBV infection [22], and cognitive decline in MS. [23] Understanding this tripartite relationship is crucial for several reasons. Firstly, cognitive dysfunction in MS does not always correlate directly with physical disability [24, 25], suggesting distinct pathogenic pathways that require independent investigation. Secondly, EBV-infected B cells in the CNS may act as persistent sources of inflammatory cytokines such as IL-6, potentially exacerbating neurodegenerative processes [26]. Thirdly, genetic variations like rs1800795 could modulate the inflammatory milieu created by EBV latency or reactivation, thereby influencing the extent of cognitive decline [27].

Recent studies have begun to illuminate the role of chronic IL-6 upregulation in cognitive disorders beyond MS, including Alzheimer's disease and other neurodegenerative conditions [28]. Elevated IL-6 levels in the CNS have been shown to impair synaptic plasticity, inhibit hippocampal neurogenesis, and promote neuronal apoptosis mechanisms that could plausibly underpin cognitive dysfunction in MS as well [28]. Thus, carriers of the rs1800795 C allele may experience amplified IL-6-driven neuroinflammatory responses following EBV infection, culminating in a higher risk or greater severity of cognitive impairment [29]. Furthermore, The rs1800795 C allele, by promoting elevated IL-6 production, might thus contribute to sustained BBB dysfunction in EBV-associated MS cases, enhancing both inflammatory infiltration and subsequent cognitive decline [30].

Genetic studies have also revealed population-specific variations in the distribution of the rs1800795 alleles, with implications for MS risk across different ethnic groups [18]. However, relatively few investigations have focused specifically on its association with disease severity or cognitive outcomes, and even fewer have contextualized these findings within the framework of EBV serostatus. Thus, dissecting the interplay between IL-6 genetic variability, EBV infection, and cognitive impairment in MS represents an important, underexplored research avenue. Moreover, targeting the IL-6 signaling pathway with therapeutic agents such as anti-IL-6 receptor monoclonal antibodies (tocilizumab) might offer novel strategies for preventing or mitigating cognitive impairment in susceptible individuals.

**Fig. 1:**
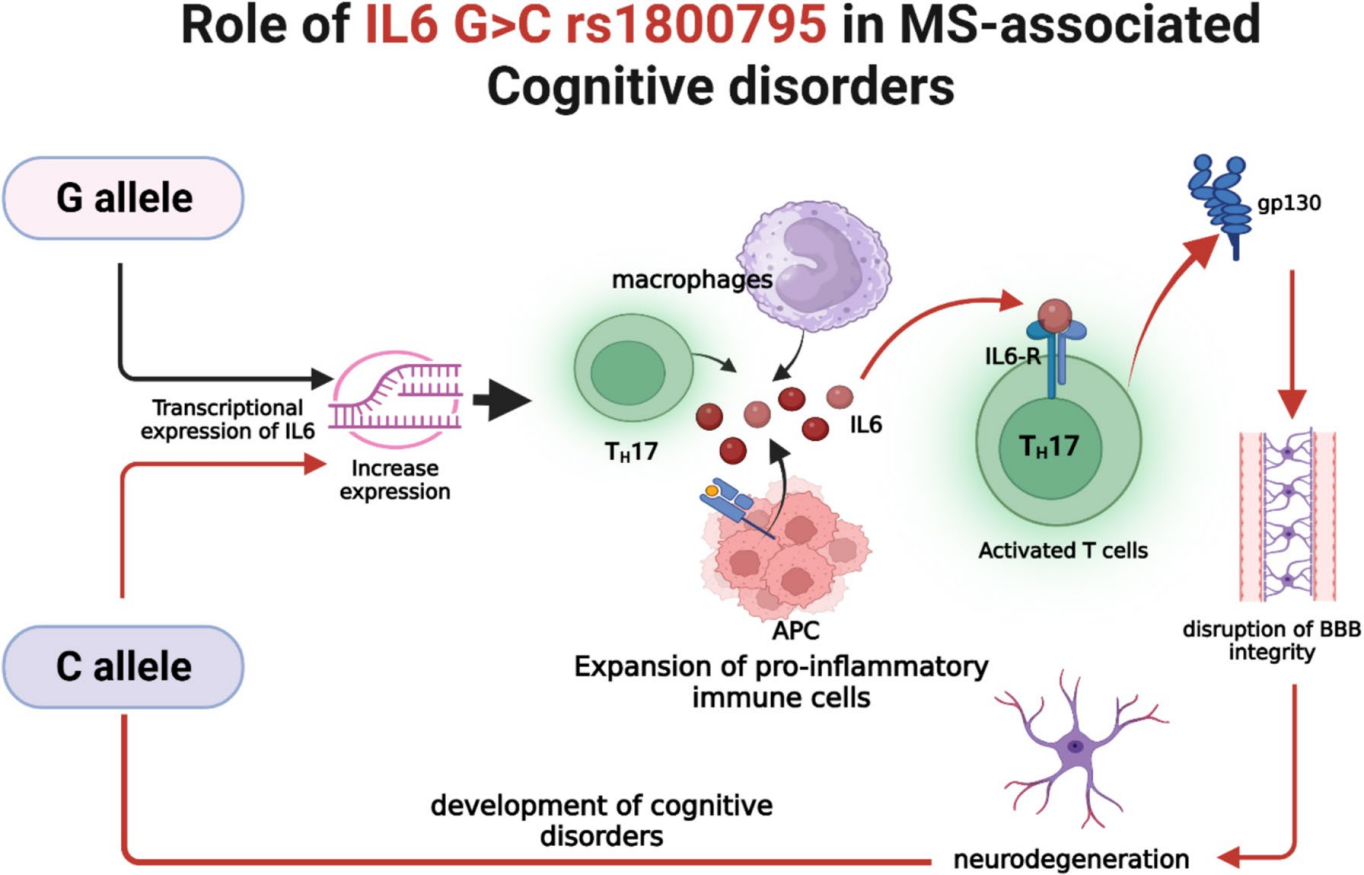
Mechanism linking the IL-6 rs1800795 C allele to cognitive impairment in EBV-associated MS. The C allele increases IL-6 production, activates gp130 signaling, enhancing neuroinflammation, Th17 activation, B-cell survival, and blood-brain barrier disruption in EBV-associated MS. This cascade leads to the activation of microglia and astrocytes, resulting in synaptic loss and neuronal damage, particularly in brain regions critical for cognition. Consequently, C allele carriers are at greater risk for cognitive impairment, supporting its role as a genetic amplifier of neurodegeneration in MS

The primary objective of this study is to investigate the association between the IL6-174 G>C rs1800795 single nucleotide polymorphism (SNP) and the severity of cognitive impairment in patients with Epstein-Barr virus (EBV) associated Multiple Sclerosis (MS). Secondary objectives include evaluating the relationship between the IL6-174 G>C rs1800795polymorphism and overall disease severity and disability progression in EBV-positive MS patients. Figure 1. Illustrates the mechanistic pathway linking the IL-6 rs1800795 C allele to cognitive impairment in EBV-associated MS.

## Methodology

### Study Design and Participants

This case-control study was conducted to investigate the association between the IL6-174 G>C rs1800795 polymorphism and susceptibility to Epstein-Barr Virus (EBV)-associated multiple sclerosis (MS). A total of 150 EBV-seropositive MS patients and 150 healthy controls matched for age and gender were recruited from a tertiary care neurology center between [June 2023 – December 2024]. MS patients were included if they met the 2017 revised McDonald criteria for MS diagnosis, confirmed by a neurologist using a combination of clinical evaluation and magnetic resonance imaging (MRI). Inclusion criteria for the MS group required participants to be seropositive for EBV (determined via EBNA1 IgG antibodies), aged between 18–55 years, and not currently undergoing immunosuppressive therapy aside from disease-modifying therapies (DMTs). Exclusion criteria included the presence of other autoimmune diseases, recent infections, malignancies, or prior immunosuppressive treatment unrelated to MS.

Healthy controls were selected from voluntary blood donors and screened for the absence of neurological disorders, EBV seropositivity, and any family history of MS or other autoimmune conditions. The study was approved by the Research Ethics Committee at the Faculty of Medicine, Ain Shams University, approval No: FMASU MS263/2024. Informed consent was obtained from all participants prior to their involvement in the study. The study was performed following all relevant ethical standards of the Declaration of Helsinki.

Disease severity was assessed using the *Expanded Disability Status Scale* (EDSS). Patients were classified into two groups based on disease severity: mild (EDSS ≤ 3) and severe (EDSS > 3), according to the criteria described by *Çinar and Yorgun* (2018) [31]. Disease progression was quantified by calculating the progression index (PI), which is defined as EDSS and divided by disease duration. Based on PI values, patients were categorized as slow progressors (PI ≤ 0.2) or rapid progressors (PI > 0.2) [32].

Moreover, Cognitive dysfunction in MS patients was confirmed using a *validated neuropsychological battery tailored for MS-related cognitive domains*. The Brief International Cognitive Assessment for MS (BICAMS) was employed as the primary tool, encompassing the Symbol Digit Modalities Test (SDMT) for information processing speed, the California Verbal Learning Test–Second Edition (CVLT-II) for verbal memory, and the Brief Visuospatial Memory Test-Revised (BVMT-R) for visual memory. All assessments were administered in a standardized, face-to-face setting by trained neuropsychologists blinded to the patient's clinical and genetic data. Raw test scores were converted into age-, sex-, and education-adjusted z-scores based on normative data. Cognitive impairment was defined as performance falling ≥1.5 standard deviations below the normative mean on at least two of the three tests, in line with international consensus criteria [33].

### Sample Size Calculation

The required sample size was calculated using a power analysis to detect a statistically significant association between the IL6-174 G>C rs1800795 variant and EBV-associated MS. Assuming a minor allele frequency (MAF) of 0.25 in the general population, an odds ratio (OR) of 1.8 based on previous studies, a significance level (α) of 0.05, and a statistical power of 80%, a minimum of 134 participants were required in each group. To ensure robustness and account for potential dropouts or genotyping failures, the sample size was increased to 150 MS patients and 150 matched healthy controls, resulting in a total of 300 participants.

### Sample Collection and Storage

Venous blood samples (5 mL) were collected into EDTA-coated tubes from all participants under aseptic conditions. Samples were immediately coded and stored at −20°C until further processing. All procedures involving human samples were handled with strict confidentiality and biosafety standards.

### DNA Extraction

Genomic DNA was extracted from peripheral blood leukocytes using the QIAamp DNA Blood Mini Kit (Qiagen, Germany) following the manufacturer's protocol. The quality and concentration of isolated DNA were assessed using a NanoDrop 2000 spectrophotometer (Thermo Scientific, USA). DNA integrity was verified by 1% agarose gel electrophoresis. All DNA samples were diluted to a working concentration of 10–20 ng/μL and stored at −20°C until further genotyping.

### Genotyping of IL6-174 G>C rs1800795 Variant

The genotyping of the IL6-174 G>C rs1800795 variant was performed using a pre-designed TaqMan SNP Genotyping Assay, cat no: 4351379, assay ID: C_1839697, C/G. Transversion substitution, Context Sequence [VIC/FAM]: [ACTTTTCCCCCTAGTTGTGTCTTGC[**C**/**G**] ATGCTAAAGGACGTCACATTGCACA], (*Thermo Fisher Scientific, USA*). The assay was carried out in a 96-well plate using the QuantStudio™ 5 Real-Time PCR System (Applied Biosystems, Thermo Fisher Scientific, USA). Each reaction contained 10 μL of TaqMan Genotyping Master Mix, 1 μL of 20× SNP Genotyping Assay Mix, 5 μL of genomic DNA (10 ng/μL), and 4 μL of nuclease-free water in a final volume of 20 μL. The thermal cycling conditions were as follows: initial denaturation at 95 °C for 10 minutes, followed by 40 cycles of denaturation at 95 °C for 15 seconds, and annealing/extension at 60 °C for 1 minute.

Validation of the genotyping assay included negative controls (no template) and replicate testing of 10% of randomly selected samples, which showed 100% concordance, confirming the reproducibility and accuracy of the assay. The allelic discrimination plots were automatically generated by the system software.

Genotype calling and allelic discrimination were performed using the Thermo Fisher Connect Cloud Software integrated with the QuantStudio Design and Analysis Software v2.6. Genotype frequencies, allelic distributions, and Hardy-Weinberg equilibrium (HWE) calculations were performed using this platform. All raw data were reviewed manually for quality assurance before statistical analysis.

### Measurement of EBV (IgG) and (IgM) antibodies

Epstein-Barr Virus (EBV) IgG and IgM antibodies were measured using commercially available chemiluminescent microparticle immunoassay (CMIA) kits provided by Abbott Laboratories. The assays were performed on the ARCHITECT i2000SR system, *Abbott Laboratories, USA*. Serum samples were processed according to the manufacturer's instructions. The ARCHITECT platform utilizes paramagnetic microparticles coated with viral antigens to capture target antibodies, followed by chemiluminescent detection. The results were expressed qualitatively as reactive or non-reactive based on the established cut-off index provided by the kit. Quality control and calibration were performed before each batch of analysis to ensure assay reliability and reproducibility.

### Statistical Analysis

Statistical analysis was conducted using IBM SPSS Statistics v26.0 (IBM Corp., USA). Descriptive statistics were reported as mean ± standard deviation (SD) for continuous variables and as frequencies and percentages for categorical variables. The chi-square test or Fisher's exact test was used to compare genotype and allele frequencies between MS patients and healthy controls. Odds ratios (OR) and 95% confidence intervals (CI) were calculated to estimate the strength of the association between the variant and MS risk. Logistic regression analysis was employed to evaluate the association between IL6-174 G>C rs1800795 genotypes and multiple sclerosis susceptibility, as well as disability severity. Odds ratios (ORs) with corresponding 95% confidence intervals (CIs) were calculated for the following comparisons: (1) GG genotype versus CG genotype between healthy controls and MS patients, (2) GG genotype versus non-GG genotypes (CC + CG combined) between healthy controls and MS patients, (3) GG genotype versus CC genotypes among MS patients stratified by Expanded Disability Status Scale (EDSS ≤5adjust for potential confounding variables such as age and gender, and GG genotype versus CC genotypes among MS patients stratified by presence or absence of cognitive disorder. A *p*-value of <0.05 was considered statistically significant.

## Results

### Demographic and Clinical Characteristics of the Studied Cohort

Table 1 presents the demographic and clinical characteristics of the studied groups. The healthy (*n*=150) and MS (*n*=150) groups were comparable in age (*p*=0.06) and gender distribution (*p*=0.168). A family history of MS was observed in 15.3% of patients. The mean disease duration in MS patients was 5.69±4.1 years. Clinically, fatigue (67.8%), pain symptoms (80.5%), and bladder dysfunction (45.6%) were common among MS patients. Vision problems were reported in 27.5%. Psychological symptoms were notable, with varying degrees of mental impairment recorded. Sensory dysfunction was observed in 59.3% of patients, ranging from mild symptoms to severe disability. Most patients had normal cerebellar function (82.6%), although some showed mild to moderate ataxia. The mean EDSS score was 3.17±1.73, indicating moderate disability, with 85% exhibiting total disability (EDSS >3). Regarding cognitive disorders, 57.7% exhibit a <1.5 standard deviation below the normative mean.

**Table 1:** Demographic & Clinical characteristics of studied groups

Variable	Healthy (*n*=150)	EBV-MS (*n*=150)	*p*-value
Age (mean±SD), range	36.31±12.38 Range: 18–62	34.63±8.39 Range: 18–53	0.06
Gender: n (%)			0.168
Male (*n*=92)	52 (34.7)	40 (26.7)	
Female (*n*=208)	98 (65.3)	110 (73.3)	
Family History: n (%)			
No		127 (84.7)	
Yes		23 (15.3)	
Duration of disease (years) mean±SD, range		5.69±4.1 Range: 1 - 24	
Fatigue n (%)			
No		49 (32.2)	
Yes		101 (67.8)	
Vision problems: n (%)			
No		108 (72.5)	
Yes		42 (27.5)	
Bladder symptoms: n (%)			
No		81 (54.4)	
Yes		68 (45.6)	
Pain symptoms: n (%)			
No		29 (19.5)	
Yes		120 (80.5)	
Cerebral (Mental) score: n (%)			
Normal		88 (59.1)	
Mood alteration only		8 (5.4)	
Mild psychological disturbance		36 (24.2)	
Moderate disturbance		15 (10.0)	
Severe disturbance with marked impairment		3 (1.3)	
Sensory Functional System Score: n (%)			
Normal		61 (40.7)	
Mild subjective symptoms (tingling, numbness)		33 (22.0)	
Mild objective signs (decreased vibration sense)		43 (28.7)	
Moderate sensory loss		6 (4.0)	
Severe sensory loss		2 (1.3)	
Severe disability or bed-bound		5 (3.3)	
Cerebellar Functional System Score: n (%)			
Normal		123 (82.6)	
Signs only (tremor, nystagmus, dysmetria)		12 (8.1)	
Mild ataxia (incoordination)		3 (1.5)	
Moderate truncal or limb ataxia		12 (1.3)	
Severe ataxia — requires support		0	
Unable to walk due to ataxia		0	
Confined to bed due to cerebellar dysfunction		0	
Expanded Disability Status Scale (EDSS) Mean ±SD (range)		3.17±1.73 Range: 1.0–6.5	
Expanded Disability Status Scale (EDSS): n (%)			
EDSS <=3: Moderate/severe disability		22 (15.0)	
EDSS > 3: Total disability		128 (85.0)	
Cognitive disorders score: n (%)			
Normal (≥1.5 SD below the normative mean)		63 (42.3)	
<1.5 below the normative mean		86 (57.7)	
Progression index (PI) (Median, range)		0.7 (0.1–0.8).	
Course of disease: n (%)			
Slow progression (PI<=0.2)		86 (57.7)	
Rapid progression (PI>0.2)		63 (42.3)	

### Distribution of IL6-174 G>C rs1800795 Genotypes and Their Association with Multiple Sclerosis

**Fig. 2:**
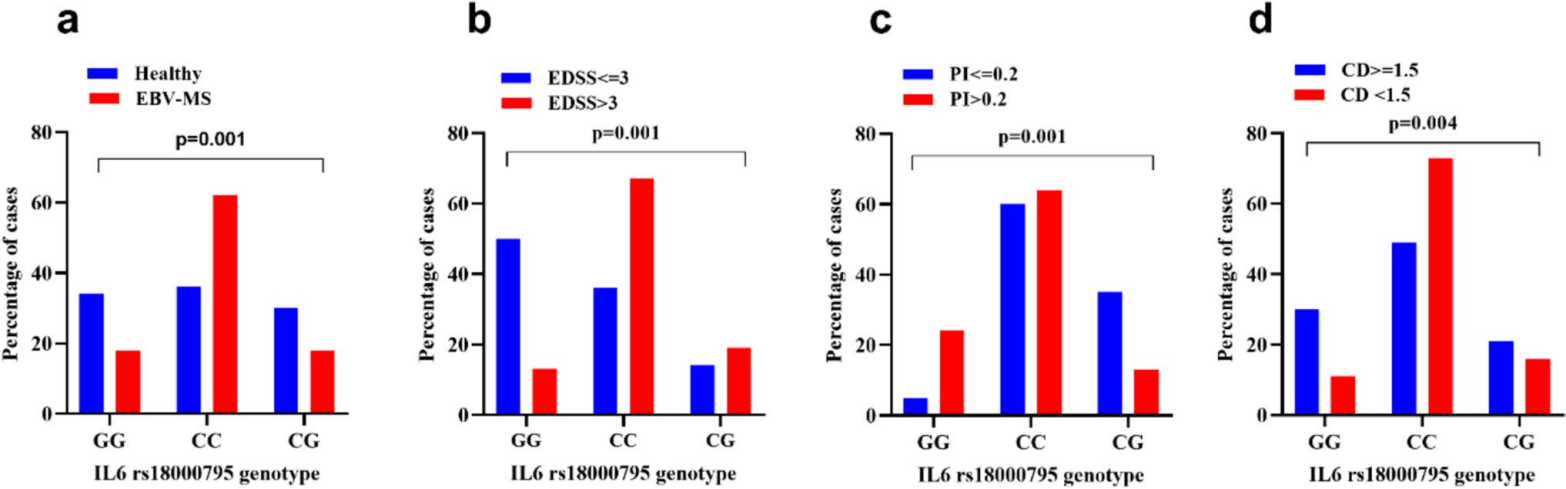
Comparative distribution of **IL6-174 G>C rs1800795** genotypes (GG, CG, CC) between healthy controls and EBV-MS patients (**a**); among EBV-MS patients with lower (EDSS ≤3) and higher (EDSS >3) disability scores (**b**); among early-based secondary progressive multiple sclerosis (EBS-MS) patients categorized by disease progression rate (**c**); Patients were divided into slow progressors (PI ≤ 0.2) and rapid progressors (PI > 0.2) (**d**); among MS patients with cognitive function >1.5 standard deviation from normal versus patients with notable cognitive dysfunction <1.5 standard deviation from normal. Data are presented as percentages of cases in each group. Statistical comparison was performed using the Chi-square test. EBV-Epstein -Barr virus, EDSS: Expanded Disability Status Scale, PI: progression index, CD: Cognitive dysfunction

Table 2 and Fig. 2a present the updated genotypic distribution of **IL6-174 G>C rs1800795** variants among healthy controls and multiple sclerosis (MS) patients. A statistically significant difference was observed between the groups (*p*=0.001, Chi-square test). The homozygous CC genotype predominated among MS patients (62.7%) compared to healthy individuals (36.0%), suggesting a strong association with increased MS susceptibility. In contrast, the homozygous GG genotype was more frequent in healthy controls (34.0%) than in MS patients (18.7%), indicating a potential protective role. The heterozygous CG genotype followed a similar pattern, being more prevalent among controls (30.0%) versus MS patients (18.7%). Figure 2a visually emphasizes the distribution shift toward CC homozygosity in the MS group.

**Table 2:** Genotypic Distribution of IL6-174 G>C rs1800795 Variants among Healthy Controls and Patients with EBV-MS

Genotype variant	Healthy (*n*=150)	MS (*n*=150)	*P*-value
Homozygous GG:79 (26.3) ^a^	51 (34.0) ^b^	28 (18.7) ^b^	
Homozygous CC: 148 (49.3) ^a^	54 (36.0) ^b^	94 (62.7) ^b^	0.001
Heterozygous CG: 73 (24.3) ^a^	45 (30.0) ^b^	28 (18.7) ^b^	

^a^: percentage is calculated within the same genotype"Homozygous CC, Homozygous GG, Heterozygous CG", ^b^: percentage is calculated within the same group"Healthy, MS", Comparative analysis was conducted using Chi-square test

### Association of IL6-174 G>C rs1800795 Genotypes with Disability Severity in EBV-MS Patients

Table 3 and Fig. 2b illustrate the distribution of **IL6-174 G>C rs1800795** genotypic variants among MS patients stratified by disability severity using the Expanded Disability Status Scale (EDSS). A significant association was observed between genotype distribution and disability level (*p*=0.001, Chi-square test). Among patients with EDSS ≤5 (moderate disability), the homozygous GG genotype was predominant (67.7%), compared to 62.7% in those with EDSS >5 (severe disability). The homozygous CC genotype was slightly more common in patients with higher disability (18.7%) compared to those with lower disability (13.4%). The Heterozygous CG genotype was less frequent overall but showed a modest increase among severely disabled patients (18.7%) versus moderately disabled (18.1%). These findings suggest that while the GG genotype is common across both disability groups, its proportion remains consistently high even with disease progression. The presence of the C allele (either CC or CG) does not seem to strongly modify disability outcomes once MS is established. Figure 2b graphically highlights the similar genotype trends across EDSS categories.

**Table 3:** Genotypic Distribution of IL6 rs1800795 (G>C) Variants among MS Patients Stratified by EDSS Score

Genotype variant	EDSS <= 3 (*n*=22, 14.8%)	EDSS >3 (*n*=128, 85.2%)	*P*-value
Homozygous GG:28 (18.8) ^a^	11 (50.0) ^b^	18 (13.4) ^b^	
Homozygous CC: 94 (63.1) ^a^	8 (36.4) ^b^	86 (67.7) ^b^	0.001
Heterozygous CG: 73 (24.3) ^a^	3 (13.6) ^b^	24 (18.9) ^b^	

^a^: percentage is calculated within the same genotype"Homozygous GG, Homozygous CC, Heterozygous GC", ^b^: percentage is calculated within the same group"EDSS <=3, EDSS >3", Comparative analysis was conducted using Chi-square test

### Genotypic Distribution of IL6 rs1800795 and Its Relationship with Disease Progression in EBS-MS

The distribution of the **IL6 rs1800795** (G>C) polymorphism was analyzed among EBS-MS patients stratified according to their disease progression index (PI) into slow (PI ≤ 0.2) and rapid (PI > 0.2) progressors. Among RP group (*n*=110), the homozygous CC genotype was more predominant (63.6%), compared to 23.6% of the GG genotype. The heterozygous CG genotype was more common among slow progressors (35%) compared to rapid progressors (12.7%). Data are presented in Table 4, Fig. 2c.

**Table 4:** Genotypic Distribution of IL6 rs1800795 (G>C) Variants among EBS-MS Patients Stratified by Progression Index (PI)

Genotype variant	Slow progression PI <=0.2 (*n*=40, 26.7%)	Rapid progression PI > 0.2 (*n*=110, 73.3%)	*P*-value
Homozygous GG:28 (18.8) ^a^	2 (5.0) ^b^	26 (23.6) ^b^	0.001
Homozygous CC: 94 (63.1) ^a^	24 (60) ^b^	70 (63.6) ^b^	
Heterozygous CG: 73 (24.3) ^a^	14 (35) ^b^	14 (12.7) ^b^	

^a^: percentage is calculated within the same genotype"Homozygous GG, Homozygous CC, Heterozygous GC", ^b^: percentage is calculated within the same progression group"Slow progression (SP) or PI <=0.2, Rapid progression (SP), PI >0.2", Comparative analysis was conducted using Chi-square test.

The distribution differences between the slow and rapid progression groups were statistically significant (*p* = 0.001), indicating a potential association between **IL6 rs1800795** genotypes and the rate of disease progression in EBS-MS patients. Notably, the higher frequency of the CC genotype among the rapid progression group suggests a possible risk for rapid disease progression

### Association Between IL6 rs1800795 Genotypes and Cognitive Dysfunction in EBV-MS Patients

Table 5 and Fig. 2d summarize the genotypic distribution of IL6 rs1800795 (G>C) variants among EBV-MS patients stratified by cognitive function. Among 149 patients, 42.3% exhibited normal cognitive performance (>1.5 SD), while 57.7% had cognitive dysfunction (<1.5 SD). The homozygous GG (wild type) genotype was more prevalent in cognitively normal patients (30.2%) compared to those with cognitive dysfunction (10.5%), suggesting a potential protective role. In contrast, the homozygous CC (mutant) genotype was significantly more frequent in cognitively impaired patients (73.3%) than in cognitively normal ones (49.2%), indicating a strong association between the CC genotype and cognitive decline. The heterozygous CG genotype showed a similar low frequency in both groups (20.6% vs. 16.3%), suggesting no clear influence on cognitive status. The differences in genotype distribution between groups were statistically significant (*p* = 0.004), supporting the association of IL6 rs1800795 polymorphism, particularly the CC genotype, with an increased risk of cognitive dysfunction in EBV-MS patients.

**Table 5:** Genotypic Distribution of IL6 rs1800795 (G>C) Variants among EBS-MS Patients Stratified by Cognitive Dysfunction (CD)

Genotype variant	Normal =>1.5 SD of normal (*n*=63, 42.3%)	Cognitive dysfunction <1.5 SD of normal (*n*=86, 57.7%)	*P*-value
Homozygous GG:28 (18.8) ^a^	19 (30.2) ^b^	9 (10.5) ^b^	0.004
Homozygous CC: 94 (63.1) ^a^	31 (49.2) ^b^	63 (73.3) ^b^	
Heterozygous CG: 73 (24.3) ^a^	14 (20.6) ^b^	14 (16.3) ^b^	

^a^: percentage is calculated within the same genotype"Homozygous GG, Homozygous CC, Heterozygous GC", ^b^: percentage is calculated within the same group"Slow progression (SP), PI <=0.2, Rapid progression (SP), PI >0.2", Comparative analysis was conducted using Chi-square test

### Logistic Regression Analysis of IL6 rs1800795 Genotypes in Relation to Multiple Sclerosis Risk and Disability Severity"

**Fig. 3:**
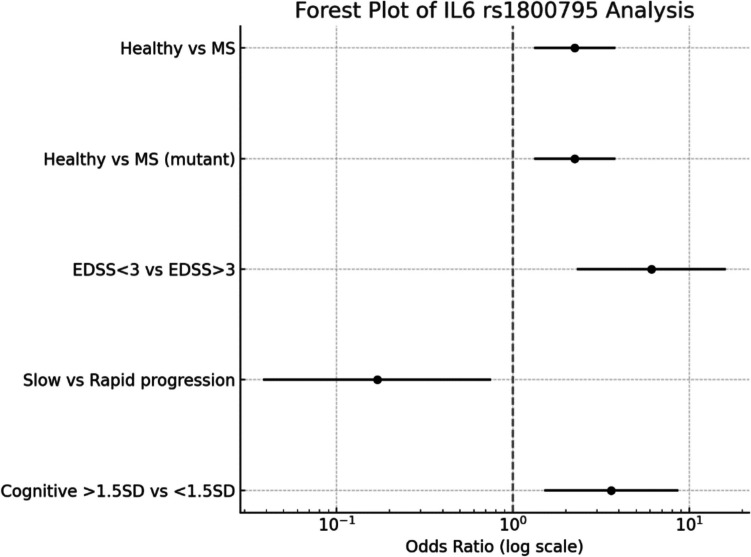
Forest plot displaying the odds ratios (OR) and 95% confidence intervals (CI) for the association between IL6 rs1800795 genotypes and various multiple sclerosis (MS) outcomes. Comparisons include healthy controls versus MS patients, disease severity measured by the Expanded Disability Status Scale (EDSS), disease progression rates based on progression index (PI), and cognitive dysfunction severity. The vertical dashed line at OR = 1.0 represents no association. Odds ratios greater than 1.0 indicate increased odds of the outcome in the comparison group, whereas odds ratios less than 1.0 suggest a protective effect. Statistical significance was determined using Fisher's exact test

In this study, we investigated the association between IL6 rs1800795 genotypes and multiple sclerosis (MS) susceptibility, disease severity, disease progression, and cognitive outcomes. The results are summarized in Table 6 and Fig. 3.

**Table 6:** Odds Ratios (OR) and 95% Confidence Intervals (CI) for the Association between IL6 rs1800795 Genotypes and MS Outcomes

Comparison	Group 1	Group 2	OR	95% CI	*P*-value
Healthy vs. EBV- MS	GG: 51 (34%) CC:54 (36%)	GG: 28 (18.7%) CC: 94 (62.7)	2.24	1.32–3.82	0.0038
Healthy vs. EBV- MS	GG: 51 (34%) CG+CC:99 (66%)	GG: 28 (18.7%) CG+CC:122 (81.7)	2.24	1.32–3.82	0.0038
EDSS <3 vs. EDSS >3	GG: 11 (50%) GC+CC:11 (50%)	GG: 18 (13.4) CG+CC:110 (86.6)	6.11	2.31–16.17	0.0004
SP vs. RP	GG:2 (5%) CG+CC:38 (90%)	GG:26 (23.6) CG+CC:84 (76.3)	0.17	0.04–0.75	0.0087
Cognitive score >1.5 SD vs. <1.5 SD	GG: 19 (30.2%) CG+CC:45 (69.8%)	GG: 9 (10.5) CG+CC:77 (89.6)	3.61	1.51–8.66	0.0052

Odds ratios (OR) were calculated using Fisher's exact test. Confidence intervals (CI) were computed at the 95% level. OR > 1 indicates higher odds in Group 1 compared to Group 2, while OR < 1 indicates lower odds, *SP* slow progressive, *RP* rapid progressive, *SD* standard deviation

#### Susceptibility to MS

Individuals carrying the CG or CC (mutant) genotypes had significantly higher odds of developing MS compared to those with the GG (wild-type) genotype (OR = 2.24, 95% CI: 1.32–3.82, *p* = 0.0038). Similarly, when comparing healthy individuals to MS patients without differentiating between CC and CG genotypes, a comparable odds ratio was observed (OR = 2.24, 95% CI: 1.32–3.82, *p* = 0.0038), reinforcing the notion that the mutant alleles are associated with increased MS risk.

#### Disease Severity

Among MS patients, those with an EDSS >3 were significantly more likely to carry the CG or CC genotypes compared to those with an EDSS score less than 3 (OR = 6.11, 95% CI: 2.31–16.17, *p* = 0.0004). This indicates that the presence of mutant alleles is strongly associated with greater neurological disability in MS.

#### Disease Progression:

When evaluating disease progression based on progression index (PI), slow progressors (PI < 0.2) were significantly less likely to carry the CG or CC genotypes compared to rapid progressors (PI > 0.2) (OR = 0.17, 95% CI: 0.04–0.75, *p* = 0.0087). An odds ratio below 1 suggests a protective effect of the GG genotype against rapid disease progression.

#### Cognitive Impairment:

Assessment of cognitive performance revealed that MS patients with cognitive scores below 1.5 standard deviations were more likely to carry the mutant alleles (CG or CC) compared to those with better cognitive outcomes (cognitive scores >1.5 SD) (OR = 3.61, 95% CI: 1.51–8.66, *p* = 0.0052). These findings suggest that the presence of the mutant IL6 rs1800795 allele may contribute to cognitive decline in MS patients. Overall, the IL6 rs1800795 CG and CC genotypes were associated with increased susceptibility to MS, greater disease severity, faster disease progression, and worse cognitive outcomes.

## Discussion

Multiple Sclerosis (MS) is a complex immune-mediated disorder influenced by both environmental and genetic factors [1]. Among these, Epstein-Barr virus (EBV) infection and inter-individual genetic variations, such as IL6 polymorphisms, have been implicated in disease susceptibility and progression [12]. Cognitive impairment, affecting a substantial proportion of MS patients, is associated with accelerated disability and highlights the need to explore genetic markers that may predict these outcomes [34]. Interleukin-6 (IL-6), a key proinflammatory cytokine, with a central role in neuroinflammation and immune regulation, has garnered increasing attention as a potential candidate gene in neuroimmune disorders [35]. Genetic factors are particularly noteworthy for their influence on influence the MS risk, severity, and cognitive outcomes [20, 36]. The novelty of this study lies in its focus on EBV-positive MS patients and its integration of immunogenetic profiling with clinical phenotyping, especially cognitive dysfunction. To our knowledge, this is the first investigation to explore the association of IL6 rs1800795 with cognitive decline in the context of EBV-associated MS, offering valuable insights into disease prediction, progression, and future immunotherapeutic targets. This study aimed primarily to investigate the association between the IL6-174 G>C (rs1800795) polymorphism and cognitive impairment in EBV-associated MS patients. Secondary objectives included exploring its relationship with overall disease severity, disability progression, and MS susceptibility.

A case-control design was adopted, including 300 subjects: 150 EBV-positive MS patients and 150 healthy controls. The MS group was further stratified by the Expanded Disability Status Scale (EDSS), progression index (PI), and cognitive dysfunction. IL6 rs1800795 genotyping was conducted using TaqMan assays. Key findings revealed a significant overrepresentation of the CC genotype among MS patients compared to controls. The CC genotype was also associated with higher EDSS scores, rapid disease progression, and greater cognitive impairment. Logistic regression further reinforced these associations, with mutant alleles (CG or CC) linked to increased odds of MS development, worsened disability, faster progression, and lower cognitive performance.

The increased frequency of the CC genotype among MS patients (62.7%) compared to controls (36.0%) and the decreased frequency of the GG genotype in the MS group (18.7% vs. 34.0% in controls) suggest that carriage of the C allele may be associated with increased disease susceptibility. These findings align with previous research linking the IL6-174 C allele with heightened IL-6 production, which is known to enhance neuroinflammatory responses and potentially exacerbate demyelination and neurodegeneration in MS. [29, 37] Conversely, the GG genotype may be protective, as evidenced by its greater prevalence in healthy individuals and patients with better cognitive outcomes [37, 38]. In contrast, the IL6 promoter region polymorphism (rs1800795) did not show a significant association with MS susceptibility in the Isfahan population, as reported by *Pourhossein et al*. [18]. This discrepancy may be attributed to differences in patient ethnicity, environmental risk factors, body composition, and sample size between their study and the current one.

When examining disease severity using EDSS, the data suggest that while the GG genotype remains common in both moderate and severe disability groups, the presence of mutant alleles (CG or CC) significantly correlates with greater disability (OR = 6.11, 95% CI: 2.31–16.17, *p* = 0.0004). This highlights a possible role for IL6 rs1800795 polymorphism not only in MS susceptibility but also in modulating the severity of neurological impairment [17]. Supporting this, recent studies have reported elevated IL-6 levels in the cerebrospinal fluid (CSF) of individuals with progressive MS, with CSF IL-6 showing positive correlations with the EDSS, the Multiple Sclerosis Severity Score, and CSF glial fibrillary acidic protein levels. These findings further underscore the potential of IL-6 as a biomarker for disease progression in MS. [39, 40]

Furthermore, disease progression analysis based on the progression index (PI) showed a higher prevalence of the CC genotype among rapid progressors (63.6%). However, the G allele, Homozygous GG (23.6%) or the Heterozygous GC (12.7%) was less frequent in rapid progressors, suggesting a potential protective effect against aggressive disease evolution. These findings underscore the possibility that IL6 genotypic variations may influence the pace of MS progression, potentially via sustained IL-6-mediated inflammation. The functional relevance of the IL6-174 G>C polymorphism in regulating transcriptional activity and elevated serum IL-6 levels has been repeatedly found in the cerebrospinal fluid (CSF) and serum of MS patients, particularly during relapses or active disease phases [18].

.Of particular interest is the significant relationship between IL6 genotypes and cognitive performance. Patients with the CC genotype were overrepresented in the cognitively impaired group (73.3%), while the GG genotype was more common among cognitively intact individuals (30.2%). Given IL-6's established role in modulating neuroplasticity, neurogenesis, and synaptic functioning [41], elevated IL-6 expression associated with the C allele may contribute to the observed cognitive deficits [37]. This is consistent with previous studies reporting IL-6's deleterious effects on hippocampal functioning and memory performance in various neurodegenerative and psychiatric disorders [29]. Our findings align with previous results, showing that homozygous CC (mutant) genotype at rs1800795 may contribute to cognitive dysfunction in MS, particularly in EBV-positive patients, highlighting a potential link between immune system adaptation and cognitive decline in disease-endemic regions. The global distribution of the IL6 −174G allele reveals distinct patterns of adaptation, particularly in regions with high infectious disease burdens. The northern migration out of Africa shows a gradual decline in the frequency of the high-producing IL6 −174G allele, with the highest frequencies in sub-Saharan Africa, which progressively decrease toward the north. This pattern is closely correlated with regional infectious disease prevalence.

In conclusion, the IL6 rs1800795 polymorphism, particularly the CC genotype, appears to be a significant genetic factor associated with increased susceptibility to MS, more severe disability, faster disease progression, and higher likelihood of cognitive impairment in EBV-positive MS patients. These associations suggest that IL6 genotyping may have future utility in prognostic stratification and personalized treatment approaches in MS management.

Despite the strengths of this study, including a well-characterized patient cohort and the integration of cognitive, clinical, and genetic data, several limitations warrant consideration. First, the study's cross-sectional design precludes definitive conclusions regarding causality. Longitudinal follow-up would be necessary to confirm the predictive value of IL6 genotypes in disease progression and cognitive decline. Second, this study focused solely on EBV-positive MS patients; while this improves cohort specificity, it may limit the generalizability of the findings to broader MS populations. Third, the unequal distribution of patients across EDSS subgroups particularly the smaller size of the EDSS <3 group compared to the EDSS >3 group may affect the reliability of subgroup comparisons and warrants cautious interpretation of these specific results. In addition, The heterogeneity of disease-modifying therapies among patients was not analyzed and may represent a potential confounding factor

It is important to note that the impact of the rs1800795 C allele on IL-6 expression remains inconsistent across different pathological conditions. This variability underscores the need for condition-specific functional analyses to clarify the allele's role in IL-6 regulation within the context of MS. Future studies should consider expanding the cohort size and including diverse MS subtypes and EBV-negative cases to validate and generalize these findings. Furthermore, integrating IL6 expression analyses, serum IL-6 measurements, and neuroimaging data may provide a more granular understanding of the pathophysiological pathways involved. Exploration of other inflammatory cytokine polymorphisms, gene-gene interactions, and epigenetic modifiers could further enhance the understanding of MS heterogeneity and cognitive impairment risk.

## Data Availability

The original data is available upon request from the corresponding author.
